# Dark Field Illumination in Diagnosis of Syphilis

**Published:** 1916-04

**Authors:** 


**Dark Field Illumination in Diagnosis of Syphilis.** R. G. Owen, Henry Snure and W. G. Pitts, Jour. Mich. State Med. Soc., Feb., tabulate 25 cases of 3-45 days' duration, 1 case urethral, 1 oral, 1 labial and 3 of finger, 19 of genitals. The dark field method showed spirochetes in 20 cases and of these only 1 reacted positively Wassermann, the oral case of 40 days' duration, though 11 others reacted positively Wassermann, 2 weeks to 6 months later. Three cases failed to show spirochetes but reacted positively Wassermann, respectively lingual, 21 days' duration, labial 22 days, and genital 15 days. Two genital cases were negative by both tests, the Wassermann being repeated at the end of 2-3 weeks and 6 weeks. These were, respectively 3 and 10 days old when first examined. How the last two cases were diagnosed is not stated, probably clinically. The superiority of the direct determination of the spirochetes, especially in the first week or so before secondary infection has occurred, as with staphylococci, and before the serum test has developed, is of practical importance, not to mention the doubt which some authors have cast on the reliability of the Wasserman test. The India ink substitute for dark field illumination may be employed but is somewhat less reliable.

